# In vitro and in vivo recombination of heterologous modules for improving biosynthesis of astaxanthin in yeast

**DOI:** 10.1186/s12934-020-01356-7

**Published:** 2020-05-12

**Authors:** Dan-Dan Qi, Jin Jin, Duo Liu, Bin Jia, Ying-Jin Yuan

**Affiliations:** 1 Frontier Science Center for Synthetic Biology and Key Laboratory of Systems Bioengineering (Ministry of Education), Tianjin University, Tianjin, 300072 China; 2 Collaborative Innovation Center of Chemical Science and Engineering (Tianjin), School of Chemical Engineering and Technology, Tianjin University, Tianjin, 300072 China

**Keywords:** In vitro recombination, In vivo recombination, Heterologous modules, Astaxanthin, *Saccharomyces cerevisiae*, Synthetic biology

## Abstract

**Background:**

Astaxanthin is a kind of tetraterpene and has strong antioxygenic property. The biosynthesis of astaxanthin in engineered microbial chassis has greater potential than its chemical synthesis and extraction from natural producers in an environmental-friendly way. However, the cost-offsetting production of astaxanthin in engineered microbes is still constrained by the poor efficiency of astaxanthin synthesis pathway as a heterologous pathway.

**Results:**

To address the bottleneck of limited production of astaxanthin in microbes, we developed in vitro and in vivo recombination methods respectively in engineered yeast chassis to optimize the combination of heterologous β-carotene ketolase (*crtW*) and hydroxylase (*crtZ*) modules that were selected from different species. As a result, the in vitro and in vivo recombination methods enhanced the astaxanthin yield respectively to 2.11–8.51 folds and 3.0–9.71 folds compared to the initial astaxanthin pathway, according to the different combination of particular genes. The highest astaxanthin producing strain yQDD022 was constructed by in vivo method and produced 6.05 mg g^−1^ DCW of astaxanthin. Moreover, it was proved that the in vivo recombination method showed higher DNA-assembling efficiency than the in vitro method and contributed to higher stability to the engineered yeast strains.

**Conclusions:**

The in vitro and in vivo recombination methods of heterologous modules provide simple and efficient ways to improve the astaxanthin yield in yeast. Both the two methods enable high-throughput screening of heterologous pathways through recombination of certain *crtW* and *crtZ* derived from different species. This study not only exploited the underlying optimal combination of *crtZ* and *crtW* for astaxanthin synthesis, but also provided a general approach to evolve a heterologous pathway for the enhanced accumulation of desired biochemical products.

## Background

**Fig. 1:**
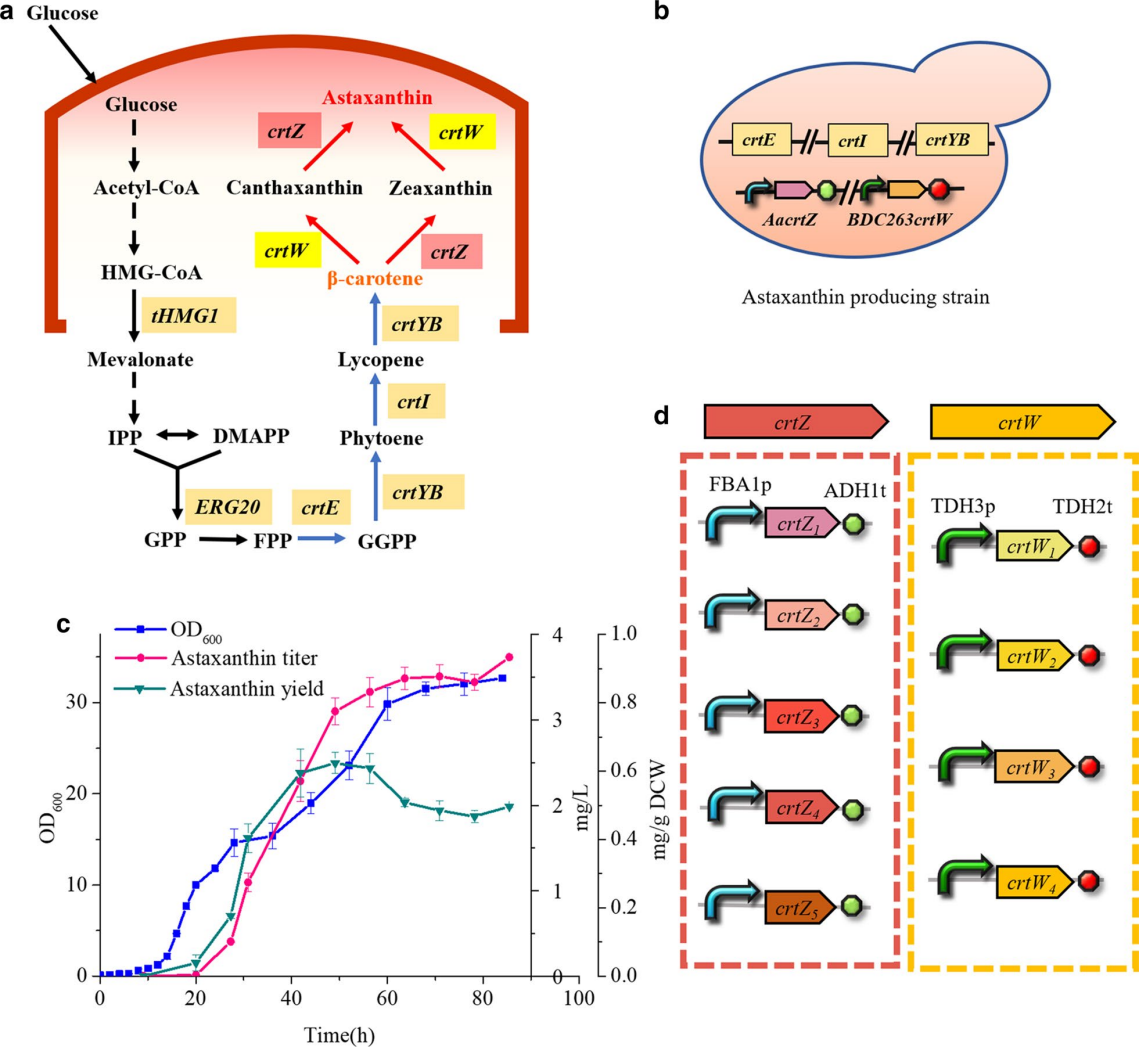
**a** Biosynthesis pathway of astaxanthin in yeast. The pathway from β-carotene to astaxanthin was boxed in the red line and engineered in this study. **b** Astaxanthin producing strain. The modular gene cassettes of *crtE*, *crtI*, *crtYB* were inserted into the CAN site of BY4741, while the *Aa. crtZ* and *B. DC263 crtW* were integrated into the retrotransposon of Ty1. **c** Profile of astaxanthin yield (green), astaxanthin titer (pink), and cell density (blue) during fermentation of astaxanthin producing strain yQDD001. **d** Sketch map of *crtZ* and *crtW* expression cassettes. Expression modules of all *crtZ*s were assembled with FBA1 promoter and ADH1 terminator. And the expression modules of all *crtW*s were assembled with TDH3 promoter and TDH2 terminator

Astaxanthin (3,3′-dihydroxy-β-carotene-4,4′-dione), a kind of carotenoid-derivative pigment with much higher antioxidant activity than other carotenoids and vitamin E [1], is commercially valuable in the aquaculture, food, cosmetic and pharmaceutical industries [2]. Traditional methods of astaxanthin production include chemical synthesis and extraction from natural producers, for example, the green algae or the red yeast [3]. However, the biosafety concerns with chemical routes and the high cost of the extraction route limits the extensive application of astaxanthin [4]. Thus far, it has been found that the astaxanthin extracted from algaes and *Paracoccus carotinifaciens* are the bioactive (3S,3′S)-stereoisomer [5, 6]. Alternatively, microbial chassis cells have been engineered for the fermentative production of astaxanthin by utilizing metabolic engineering techniques [7]. This way has become a promising alternative to produce terpene-derivatives that meet the safety and economic concerns. Modifying microbial cells to improve the production of the desired endogenous or exogenous metabolites is a broad aim at many areas of academic, industrial biotechnology and biosciences [8–11]. In recent years, the heterologous biosynthesis of astaxanthin has been successfully achieved in *Escherichia coli* [12–17], *Saccharomyces cerevisiae* [5, 18–20], *Yarrowia lipolytic* [21], and *Corynebacterium glutamicum* [22] by introducing particular biosynthesis pathways. However, the astaxanthin yields in these engineered microbes were still not high enough for cost-worthy commercialization. The total biosynthesis pathway of astaxanthin in yeast is complex and full of branches, as shown in Fig. 1a. Glucose is converted into farnesyl pyrophosphate (FPP; C15) through the glycolytic pathway and mevalonate (MVA) pathway, and FPP is converted into β-carotene by the reaction of *crtE*, *crtYB* and *crtI*. The final synthesis of astaxanthin from β-carotene is a metabolic web containing several branches, according to the different participation steps and orders of the β-carotene ketolase (*crtW*) and hydroxylation (*crtZ*) [23]. It has been revealed that many bacterial *crtZ*s and *crtW*s could utilize β-carotene as well as its hydroxylated or ketonic products as the substrate, leading to diverse carotenoid intermediate profiles which can greatly affect astaxanthin yield and ratio [24–26]. Choi et al. has reported that a combination *crtW* from *Brevundimonas* sp. SD212 (*BSD212_crtW*) and *crtZ* from *Erwinia uredovora* (*Eu_crtZ*) generated more astaxanthin and fewer hydroxylated intermediates than the combination of *crtW* from *Paracoccus* sp. N81106 (PN81106_*crtW*) and Eu_*crtZ*, probably due to substrate preference for none-ketonic carotenoids [27]. Meanwhile, it has also been reported that by integrating *crtW* from *Brevundimonas vesicularis. DC263* and *crtZ* from *Alcaligenes* sp. strain PC-1 into a β-carotene producing strain, higher astaxanthin yield was achieved in *S. cerevisiae* via ketylation first and hydroxylation subsequently [20]. The combination of *crtZ* and *crtW* from different species is still critical for the enhanced astaxanthin accumulation.

In this study, we report the in vitro recombination and the in vivo recombination methods for improvement of astaxanthin yield in yeast. The in vitro method is using the in vitro Cre/LoxP recombination system to screen the heterologous modules of *crtW* and *crtZ* in CEN/ARS plasmids followed by yeast transformation. The in vivo method is using yeast homologous recombination system (HR) to directly integrate the heterologous modules of *crtW* and *crtZ* into the Ty1 retrotransposon sites in the yeast genome. The yeast with the highest astaxanthin yield in this study was obtained by in vivo recombination. Our study demonstrated that the transformation efficiency of in vivo recombination was higher than in vitro recombination. Moreover, in vivo recombination strain was more stable than the in vitro recombination strain. These results indicate that the in vivo combination of heterologous pathway modules has higher efficiency and stability than the in vitro recombination. The combination of heterologous pathway modules of *crtW* and *crtZ* is useful for fine-tuning of metabolic flux, which significantly increased the yield of astaxanthin up to 9.71-fold compared to the ancestor strain, highlighting the use of our strategy.

## Results and discussion

### Construction of astaxanthin producing strain

According to the previous studies in our lab, combination of *crtZ* from *Agrobacterium aurantiacum* (*Aa crtZ*) and *crtW* from *Brevundimonas vesicularis DC263* (B. DC263 *crtW*) has a positive impact on the astaxanthin pathway in yeast [19, 20]. Thus, *Aa crtZ* and *B. DC263 crtW* were chosen to construct the initial astaxanthin-producing strain. The high β-carotene producing strain yQDD000, with carotenoid biosynthesis pathway (*crtE*, *crtI*, and *crtYB* from the carotenoid-producing yeast *Xanthophyllomyces dendrorhous* with Leu2 marker) integrated into the CAN sites of yeast strain BY4741, could provide amounts of substrate for the synthesis of astaxanthin. The yQDD001 was constructed through the co-integration of the *Aa crtZ* and *B. DC 263 crtW* accompanied with the G418 marker integrated into the Ty1 retrotransposon of yQDD000 (Fig. 1b). The HPLC detection of the carotenoid products extracted from the strain yQDD000 and yQDD001 was shown in additional Fig. 1. The β-carotene producing strain showed an onefold β-carotene peak at 20.2 min, while strain yQDD001 showed astaxanthin peak at 6.4 min along with other peaks of the identified intermediates, such as zeaxanthin (IV) at 7.5 min, canthaxanthin (III) at 10.5 min and lycopene (II) at 18.3 min. The profile of astaxanthin yield, astaxanthin titer, and cell density during the fermentation of the strain yQDD001 in the 50 mL flask was shown in Fig. 1c. Eventually, a yield of 0.623 mg g^−1^ DCW astaxanthin was obtained after 44 h cultivation. To further optimize the heterologous pathway, we selected five alternative *crtZ* from five other species and four *crtW* from four species to test whether their participation into the pathway would improve astaxanthin production (Table 1). We located one pair of the promoter FBA1p and terminator ADH1t for the expression of *crtZ*s and another pair of TDH3p and TDH2t for the expression of *crtW*s (Fig. 1d). We designed nine PCR tags for screening each gene of *crtZ*s or *crtW*s by inserting the tags downstream of each gene’s terminator (Additional file 1: Table S1).

**Table 1:** Heterologous modules *crtZ* and *crtW* used in this study

Name of *crtZ/crtW*	Microbial source	Called in this study
*Aa crtZ*	*Agrobacterium aurantiacum*	*crtZ*_*1*_
*B. DC263 crtZ*	*Brevundimonas vesicularis DC263*	*crtZ*_*2*_
*B. SD212 crtZ*	*Brevundimonas* sp. *SD212*	*crtZ*_*3*_
*HpChyb crtZ*	*Haematococcus pluvialis*	*crtZ*_*4*_
*SsP2 crtZ*	*Sulfolobus solfataricus P2*	*crtZ*_*5*_
*Aa crtW*	*Agrobacterium aurantiacum*	*crtW*_*1*_
*Asp crtW*	*Alcaligenes* sp. strain	*crtW*_*2*_
*B. DC263 crtW*	*Brevundimonas vesicularis DC263*	*crtW*_*3*_
*GvccrtW*	*Gloeobacter violaceus PCC 7421*	*crtW*_*4*_

### In vitro recombination of heterologous modules *crtZ* and *crtW*

**Fig. 2:**
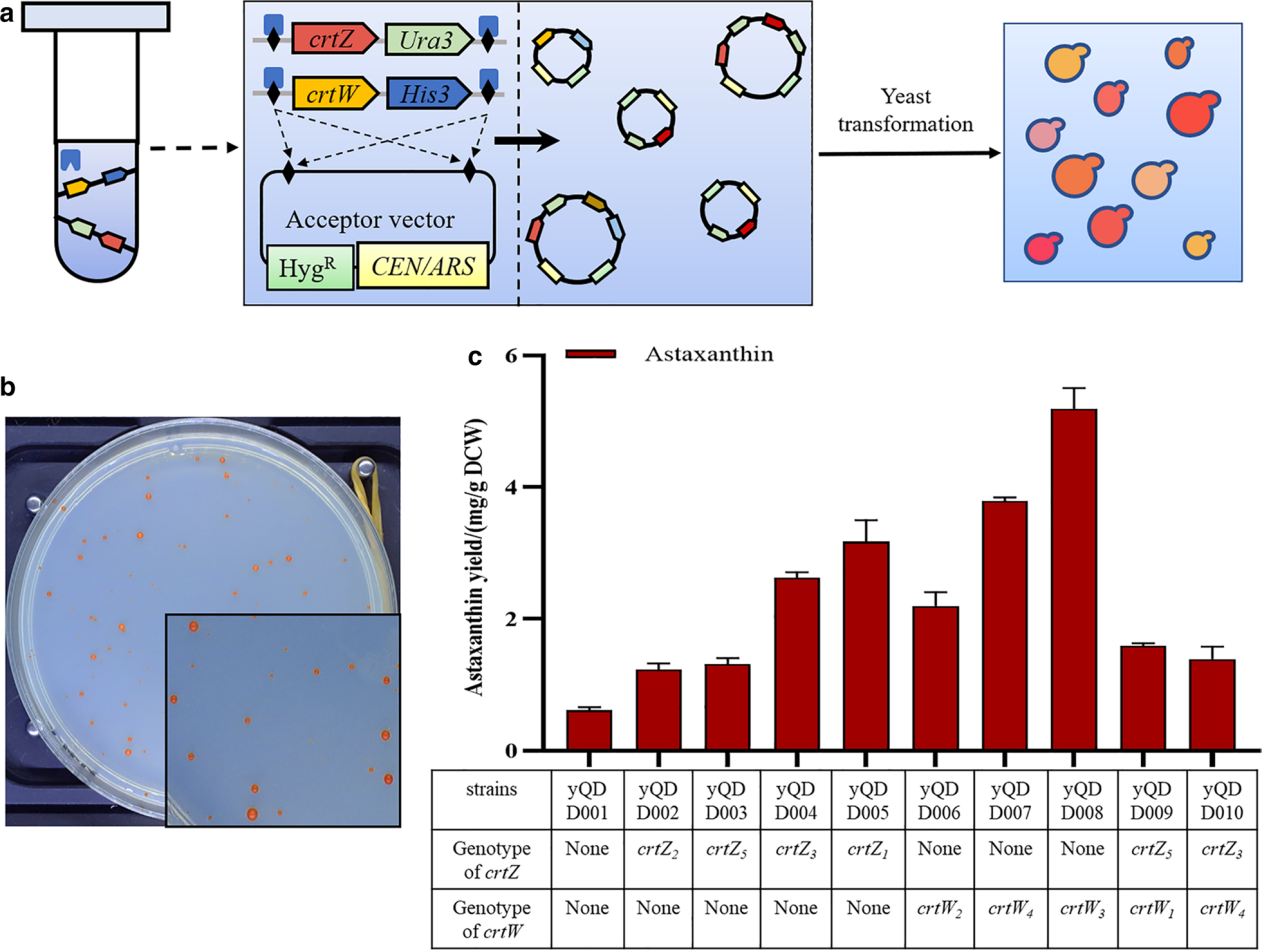
In vitro recombined of heterologous modules *crtZ* and *crtW*. **a** Workflow of the in vitro recombination to evolve the heterologous pathway of astaxanthin in yeast. The acceptor vector and the pool of donor constructs (*crtZ* and *crtW*) are mixed with Cre recombinase in vitro. The donor fragments will be randomly inserted into LoxPSym sites of the acceptor vector, assembled into various new plasmids. **b** The reaction products were transformed into yQDD001, and produced the yeast library with different colors and sizes. **c** Astaxanthin yield measurement of in vitro recombined strains. And the genotype of yQDD002–yQDD010 was proved by PCRTag analysis. The error bars represent standard deviations calculated from duplicate experiments. “Astaxanthin yield” was determined as “the astaxanthin content in single-cell” with the unit as mg g^−1^ DCW

To further improve the yield of astaxanthin in yQDD001, in vitro recombination was used to rearrange the heterologous genes of astaxanthin in vitro [28]. Cre/LoxP was a widely used site-specific DNA recombination system derived from bacteriophage P1. The LoxP site was 34 bp in length, consisting of two 13 bp inverted repeats separated by an 8 bp asymmetric spacer sequence. Cre recombinase catalyzed a site-specific recombination reaction to two LoxP sites and did not require accessory factors. Concerning the molecular mechanism of recombination, a single recombinase molecule binded to each palindromic half of LoxPSym sites, then the recombinase molecules formed a tetramer, thus bringing two LoxP sites together [29]. Depending on the direction of LoxP, the Cre/LoxP system could be used to generate deletions, inversions, insertions (transpositions), or translocations. If LoxP sites encoded a symmetric spacer region (LoxPSym), rearrangements were orientation-independent between two LoxPSym sites. The in vitro recombination method specified the use of Cre recombinase for rearrangement of *crtZ* and *crtW* expression constructs each containing a pair of LoxPSym sites besides the gene’s transcriptional unit along with an accompanied *Ura3*/*His3* marker (Fig. 2a). The in vitro recombination started with a centromeric acceptor vector and a series of candidate genes (*crtZ*s and *crtW*s, represented as “donor fragments”). In addition, the acceptor vector encoded a hygromycin resistance gene (represented as Hyg^R^). Tow LoxPSym sites were located in the outsides of the cassettes of Hyg^R^ and CEN/ARS in the acceptor vector. The acceptor vector was digested by *EcoR*I and *Bam*HI and the linearized fragment was purified for the preparation of in vitro recombination reaction. The donor fragments were generated by digestion of *Not*I and *Xba*I from their pUC19-based plasmids. During this study, all the donor fragments of *crtZ* and *crtW* were mixed with acceptor vector as the reaction pool of in vitro recombination. The donor fragments were recombined with the acceptor vector randomly under the action of Cre recombinase and produced a pool with diverse plasmids (Additional file 1: Figure S2). Then the plasmids pool was transformed into yQDD001 for generating the yeast colony library which presented different colors and sizes (Fig. 2b). The selection marker for *His*^+^/*Ura*^+^ and Hyg^R+^ were used to make sure at least one or two donor fragments were recombined into the acceptor vector during the in vitro recombination reaction. There were about 100 colonies appeared on the plates after yeast transformation. We picked almost every colony to screen for the potential highest astaxanthin producing strain. According to the visual judgement, darker red colonies were selected from the whole colonies. Finally, there were nine darker-red colonies (yQDD002–yQDD010) were optimally selected and the astaxanthin tier in these strains were detected by HPLC. The recombination plasmids were verified by PCR-Tag analysis and sequencing. The copy number of *crtZ* and *crtW* fragments in recombined plasmids was analyzed by qPCR (Additional file 1: Figure S3). The results indicated that there was only one copy number of *crtZ* or *crtW* in the yQDD002–yQDD010. As shown in Fig. 2c, astaxanthin yields of yQDD002–yQDD010 were increased to 1.24, 1.32, 2.63, 3.18, 2.20, 3.79, 5.50, 1.59 and 1.39 mg g^−1^ DCW, respectively. The in vitro recombination strains increased the astaxanthin yield by 1.98- to 8.51-fold compared with yQDD001. The results demonstrated that the additional heterologous genes of *crtZ* or *crtW* had positive effect on the astaxanthin synthesis in yeast.

### In vivo recombination of heterologous modules

**Fig. 3:**
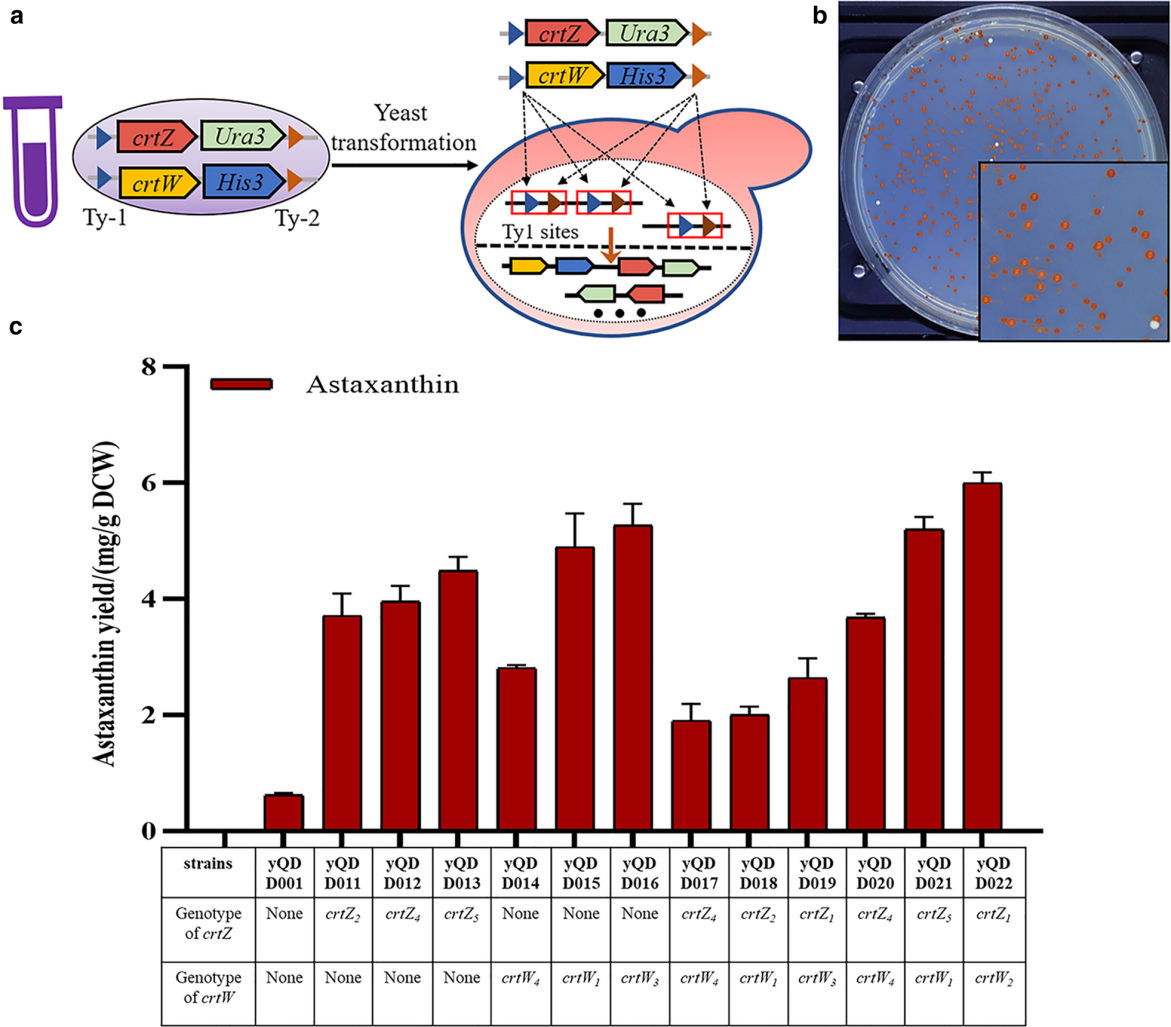
In vivo recombination of heterologous modules. **a** Each fragments *crtZ*/*crtW* carry an *Ura3*/*His3* marker and two homologous arms with Ty1 (Ty-1, Ty-2). All *crtZ* and *crtW* fragments were mixed up to transform into yQDD001. The fragments inserted into the Ty1 sites of yeast genome randomly to produce the yeast library with various combinations of *crtZ* and *crtW*. **b** Yeast colonies of in vivo recombination method screened in the selected medium. The yeast library contained various yeast with different colors and sizes. **c** Astaxanthin yield measurement of in vivo recombination evolved strains. The genotype of yQDD011–yQDD022 were proved by PCRTag analysis

Guided by the industrial experience of microbial fermentation, it was supposed that the high stability of the recombined heterologous pathway could increase astaxanthin accumulation in yeast. The plasmids constructed by in vitro recombination might be lost during the fermentation process with YPD medium,which might lead to the reduction of astaxanthin accumulation. There were multiple Ty1 retrotransposon sites in yeast genome [30], which could be used for the integration of multiple copies of heterologous modules by in vivo recombination methods. To integrate the *crtZ* and *crtW* into the genome, all the *crtZ* and *crtW* were flanked by about 500 base pair homologous sequences selected from the TyA (Additional file 1: Figure S4). As shown in Fig. 3a, the integration cassettes of all *crtZ* and *crtW* were mixed and transferred into the yQDD001 (Additional file 1: Figure S5). As shown in Fig. 3b, the in vivo recombination method generated the yeast library with various color and size colonies. Finally, the darker red colonies (yQDD011–yQDD022) from in vivo recombination were selected for characterization and their production of astaxanthin was detected by HPLC. The particular specie-derived genes of *crtZ* or *crtW* randomly inserted into the yeast genome were identified by the designed PCRTag analysis (Additional file 1: Table S1). As shown in Fig. 3c, the astaxanthin yields in strain yQDD011–yQDD022 were increased to 3.71, 3.96, 4.49, 2.81, 4.89, 5.26, 1.90, 2.00, 2.63, 3.67, 5.20, and 6.05 mg g^−1^ DCW, respectively. The in vivo recombination strains increased astaxanthin yield 3.05- to 9.71-fold compared with the yQDD001, respectively. And the genotypes of *crtZ*/*crtW* were listed in Fig. 3c. As shown in Additional file 1: Figure S6, the copy number of *crtZ* and *crtW* of the yQDD011 to the yQDD022 were assayed by qPCR. According to the astaxanthin yield in selected strains the combination of *crtZ*_*1*_ and *crtW*_*2*_ had much more positive effects than other combinations on the astaxanthin pathway in the host strain. The increase in *crtZ* and *crtW* copy numbers had a positive impact on the astaxanthin pathway in the host strain. There were two copies of *crtZ*_*4*_ in yQDD017, while only one copy of *crtZ* or *crtW* in other strains. It was noted that the astaxanthin yield of yQDD017 was lower than that of yQDD020, which contained one copy of *crtZ*_*4*_ and *crtW*_*4*_. The astaxanthin yield of yQDD012 was higher than yQDD017 and yQDD020, which had only one *crtZ*_*4*_ but no *crtW*. These results indicated that the overexpression of *crtZ* or *crtW* might have a negative impact on astaxanthin yield. The astaxanthin yield comparison between yQDD011–yQDD015–yQDD018 also indicated that. This might relate to the increased metabolic burden caused in the increase of *crtZ*/*crtW* copy number. Otherwise, the mutation in the genome caused by the integration of *crtZ* and *crtW* might induce astaxanthin metabolic changes. There were multiple Ty1 retrotransposon sites in the yeast genome. However, the number of strains with single copy integration was much more than the strains with multiple copies integration without any other environment pressure. It was helpful to truncate the promotor of selection marker [31] or apply the Di-CRISPR [32] to increase the integration efficiency. Combination of those methods may further to improve the efficiency of our in vivo recombination methods.

### Efficiency analysis and stability analysis of heterologous modules

For the in vitro recombination method, different *crtZ* and *crtW* fragments with LoxPSym sites were assembled to new plasmids in tubes by the in vitro Cre/LoxP system, and the new recombined plasmids were transformed into the host strain yQDD001. For the in vivo recombination method, the different *crtZ*/*crtW* fragments with the homologous arm of Ty1 sites were transformed into the host strain, and the *crtZ* and *crtW* integrated into the Ty1 retrotransposon sites by homologous recombination. Both the in vitro and in vivo recombination method could screen *crtZ* and *crtW* derived from different species randomly and could be used for the accumulation of natural products in the microorganism. In this study we worked on the efficiency analysis and stability analysis of heterologous modules in these two methods.

The colony’s number of the in vitro screening and the in vivo screening followed yeast transformation with three biological repeats were used to assess the efficiency of in vitro and in vivo recombination. All the experimental conditions remained consistent, including the concentration of DNA fragment, the biomass of host strain yQDD001 and other operating environments. Photograph of the in vitro and the in vivo screening was attached. The colony’s number was listed in Additional file 1: Figure S7. There were about 100 single colonies under the in vitro screening after yeast transformation while there were about 550 single colonies under the in vivo screening. Compared with in vitro recombination, more colonies appeared on the plates after in vivo recombination method, indicating the higher transformation efficiency of the in vivo recombination.

**Fig. 4:**
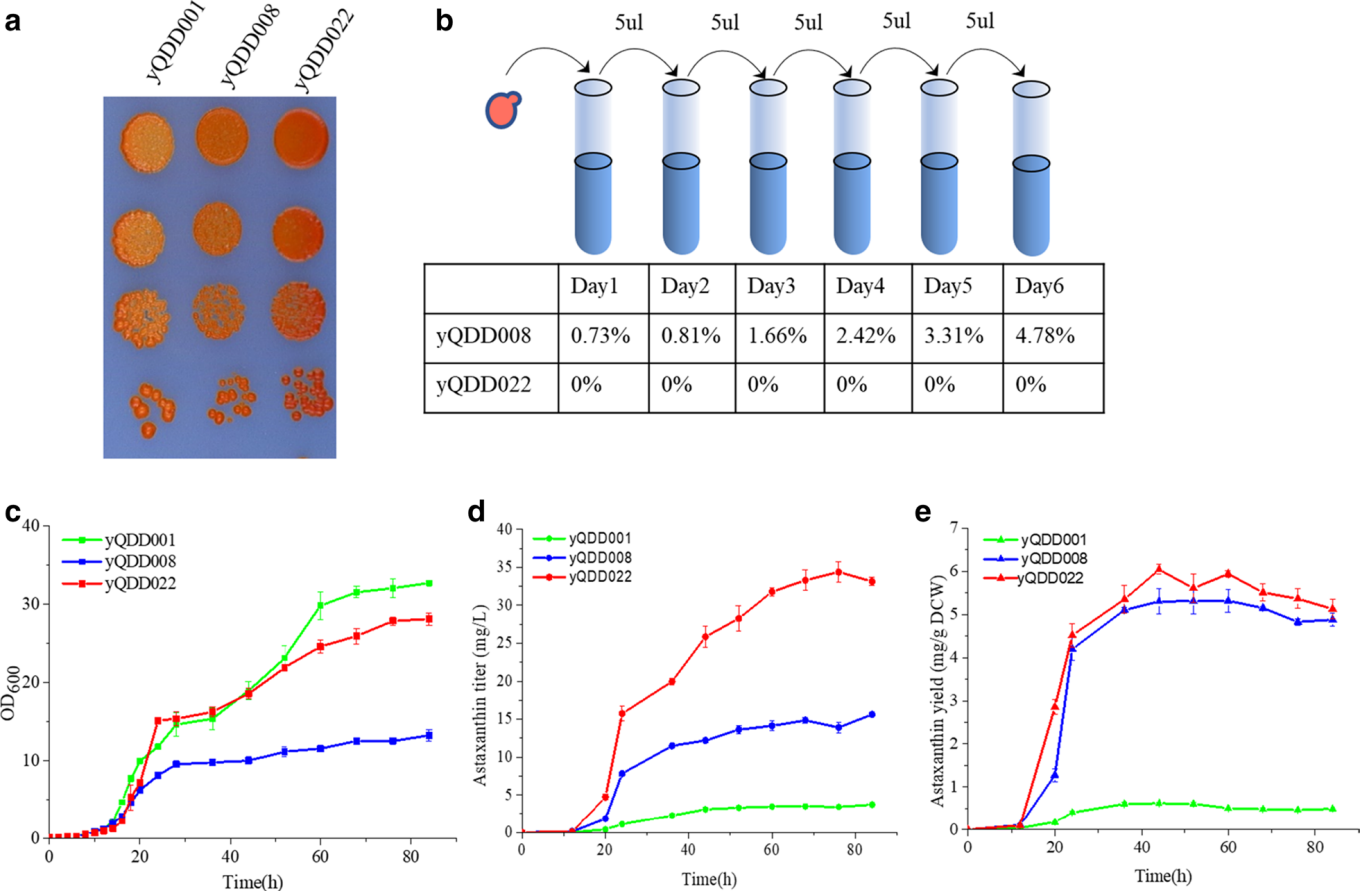
The comparison between the in vitro and the in vivo evolution of heterologous modular pathway of astaxanthin. **a** Phenotype verification of yQDD008 and yQDD022. The parent strain yQDD001 was used as control strain. The photograph was attached to illustrate the visual color of the related strains. **b** Stability assays of yQDD008 and yQDD022. Yeast cultures in YPD after 6 days were plated on SD agar and the number of light-color strains was counted. The ratio of unstable strains in every 12 generations was recorded and listed. **c** Growth curves of yQDD001, yQDD008 and yQDD022. **d** Profile of astaxanthin titer during fermentation with strain yQDD001, yQDD008 and yQDD022. **e** Profile of astaxanthin yield during fermentation with strain yQDD001, yQDD008 and yQDD022

The strains yQDD008 and yQDD022, which performed the highest astaxanthin yield from the in vitro and the in vivo recombination respectively, were selected for characterizing the growth and stability between these two methods. Ten-fold serial dilutions of yQDD008 and yQDD022 were spotted on the SD agar plates. yQDD001 was used as control. As shown in Fig. 4a, yQDD008 and yQDD022 display darker red than the parent strain yQDD001, indicating the higher astaxanthin production. The yQDD008 and the yQDD022 were serially subculture in YPD for 6 days and then spotted on SD solid medium each day (Additional file 1: Figure S8). The colonies of light color were observed in the screened medium of yQDD008, while not observed in yQDD022. The ratio of colonies with light color was listed in Fig. 4b. This result proved the high stability of heterogeneous pathway recombined by in vivo method. However, compared with yQDD001, the increase of *crtZ* and *crtW* copy number had negative effects on yeast growth (Fig. 4c). The profile of astaxanthin titer and astaxanthin yield during fermentation in the 50 mL flask with strain yQDD001, yQDD008 and yQDD022 were shown in Fig. 4d, e. The astaxanthin yield reached maximal value at 52 h (yQDD008 with 5.30 mg g^−1^ DCW) and 44 h (yQDD022 with 6.10 mg g^−1^ DCW). And the glucose consumption profile of two strains was similar (Additional file 1: Figure S9). The low stability of recombined plasmids by in vitro method may have a negative impact on the accumulation of astaxanthin in yQDD008. These results indicated that the stability of strains obtained by in vivo recombination is higher than that by the in vitro recombination. And the highest astaxanthin producing yeast strain was obtained by in vivo recombination of *crtZ*_*1*_ and *crtW*_*2*_. This method can increase the astaxanthin yield of yeast significantly in a high throughput way. The in vivo recombination method has a great potential to increase the efficiency and copy numbers of heterologous modules in the yeast genome.

## Conclusion

In this study, the exogenous genes of *crtZ* and *crtW* derived from different species were combined randomly in astaxanthin producing strain by in vitro and in vivo method. The results indicated that the increase of *crtZ* and *crtW* copy numbers have a positive impact on the improvement of astaxanthin yield in yeast. Compared with in vitro recombination, the in vivo recombination method showed higher integration efficiency and higher stability of the heterologous modules. Finally, the strain yQDD022 with the highest yield of astaxanthin (6.05 mg g^−1^ DCW) in this study was obtained by in vivo recombination, which with the integration of *crtW* and *crtZ* from *Alcaligenes* sp. strain and *Agrobacterium aurantiacum*, respectively.

Traditional genetic manipulation costs lots of time to construct the heterologous pathway to screening different sources of *crtZ*s and *crtW*s. However, both the in vivo and in vitro recombination method could produce a variety combination of *crtZ* and *crtW* in a short time. The advantages of the in vitro recombination method are fast and easy to obtain the genotype of heterologous genes which are responsible for the phenotype variation, without limitations of the host strains. The advantages of the in vivo recombination method are more stable for constructing and screening the heterologous pathway at the same time, which can be used for industry strains metafiction directly. Both the in vitro and in vivo recombination research could provide reference to the recombination of heterologous pathway in building microbial cell factories. This study has great reference values to improve the accumulation of desired compounds in yeast with efficiency and stable performance.

## Methods and materials

### Strains and media

Yeast strains used in this study were described in Table 2. The astaxanthin producing strain yQDD001 (*MATa*, *His3*Δ0, *Leu2*Δ1, *met15*Δ0, Ura*3*Δ0, *HO::tR(ccu)J*, *lys::NAT*) was subjected to improve the host’s compatibility with *crtZ* and *crtW* from different sources by in vitro and in vivo recombination. Selective medium for rearrangement strains were SC-Leu-Ura + G418 (synthetic complete medium lacking leucine and uracil with 20 g L^−1^ glucose and 100 μg mL^−1^ G418), SC-Leu-His + G418 (synthetic complete medium lacking leucine and histidine with 20 g L^−1^ glucose and 100 μg mL^−1^ G418) and SC-Leu-Ura-His + G418 (synthetic complete medium lacking leucine, uracil, and histidine with 20 g L^−1^ glucose). All yeast solid media were added with 20 g L^−1^ agar. *Escherichia coli* DH5α purchased from BEIJING Biomed Co., Ltd was used for plasmid transformation. *Escherichia coli* were cultivated at 37 °C in LB medium (with 10 g L^−1^ tryptone, 5 g L^−1^ yeast extract, 10 g L^−1^ NaCl and 100 μg mL^−1^ ampicillin). LB solid medium was added with 15 g L^−1^ agar.

**Table 2:** Strains used in this study

Strains and plasmids	Description	Sources
yQDD000	By4741 with a carotenoid pathway (Leu2 Marker) into the YEL063C/CAN1 locus in chromosome V	This lab
yQDD001	yQDD000 with *Aa crtZ* and *B. DC 263 crtW* (with the G418 marker) were integrated into the retrotransposition of Ty1	This study
yQDD002	In vitro recombined strain from yQDD001 with *crtZ*_*2*_	This study
yQDD003	In vitro recombined strain from yQDD001 with *crtZ*_*5*_	This study
yQDD004	In vitro recombined strain from yQDD001 with *crtZ*_*3*_	This study
yQDD005	In vitro recombined strain from yQDD001 with *crtZ*_*1*_	This study
yQDD006	In vitro recombined strain from yQDD001 with *crtW*_*2*_	This study
yQDD007	In vitro recombined strain from yQDD001 with *crtW*_*4*_	This study
yQDD008	In vitro recombined strain from yQDD001 with *crtW*_*3*_	This study
yQDD009	In vitro recombined strain from yQDD001 with *crtZ*_*5*_ and *crtW*_*1*_	This study
yQDD010	In vitro recombined strain from yQDD001 with *crtZ*_*3*_ and crt*W*_*4*_	This study
yQDD011	In vivo recombined strain from yQDD001 with *crtZ*_*2*_	This study
yQDD012	In vivo recombined strain from yQDD001 with *crtZ*_*4*_	This study
yQDD013	In vivo recombined strain from yQDD001 with *crtZ*_*5*_	This study
yQDD014	In vivo recombined strain from yQDD001 with *crtW*_*4*_	This study
yQDD015	In vivo recombined strain from yQDD001 with *crtW*_*1*_	This study
yQDD016	In vivo recombined strain from yQDD001 with *crtW*_*3*_	This study
yQDD017	In vivo recombined strain from yQDD001 with *crtZ*_*4*_ and *crtW*_*4*_	This study
yQDD018	In vivo recombined strain from yQDD001 with *crtZ*_*2*_ and *crtW*_*1*_	This study
yQDD019	In vivo recombined strain from yQDD001 with *crtZ*_*1*_ and *crtW*_*3*_	This study
yQDD020	In vivo recombined strain from yQDD001 with *crtZ*_*4*_ and *crtW*_*4*_	This study
yQDD021	In vivo recombined strain from yQDD001 with *crtZ*_*5*_ and *crtW*_*1*_	This study
yQDD022	In vivo recombined strain from yQDD001 with *crtZ*_*1*_ and crt*W*_*2*_	This study

### Yeast transformation and assembly

The protocol used for yeast transformation is the LiAc/SS carrier method. Yeast colonies were inoculated into 5 mL of SC-Leu + G418 and grown overnight at 30 °C. Then 200 μL yeast solution was inoculated into 5 mL of new SC-Leu + G418 cultures. 5–6 h after, cultures were washed out twice with ddH_2_O (double-distilled water) and resuspended in 0.1 M LiAc put on ice until needed. Yeast transformation system contained 620 μL of 50% polyethylene glycol (PEG) with molecular weight 3350, 40 μL salmon sperm DNA (SSDNA, 100 mg mL^−1^), 90 μL of 1 M LiAc solution. Then, 50 μL in vitro or in vivo recombination system was mixed with 100 μL resuspended cells. And the mixed pool was added into LiAc/SS carrier DNA/PEG mixture and stir spirally. Samples were first incubated at 30 °C for 30 min. Then heat-shocked 18 min at 42 °C water-bath. 90 μL DMSO was added followed by heat-shocked. Centrifuged and resuspended cells with 400 μL 5 mM CaCl_2_, plated on selective medium after 10 min. After culturing for 72 h at 30 °C incubator, darker red yeast colonies were selected on synthetic medium.

### In vitro recombination

As shown in Additional file 1: Figure S2, the donor fragments and acceptors were cut from the plasmids by the enzyme. The 50 μL reaction system of in vitro recombination contained 1000 ng acceptor vectors, the donor fragments pool of *crtZ* and *crtW* from different sources (1000 ng, respectively) and 2 μL of high concentration Cre recombinase (NEB, M0298M). Refer to previous studies of Zhu et al. [28], the Cre recombinase reaction was set up as incubated at 37 °C for 4 h. The Cre enzyme was heat-inactivated for 10 min at 70 °C. Then the reaction pools were transformed into hosts strains yQDD001 for genotype and phenotype testing. SC-Leu-Ura + G418, SC-Leu-His + G418 and SC-Leu-Ura-His + G418 medium were used to select for recombined constructs. To ensure the quality of color screening, all the yeast transformation was diluted fivefold and repeated three times in this study. There are thousands of colonies generated for screening high producing strains.

### In vivo recombination

As shown in Additional file 1: Figure S5, The fragments of *crtZ* and *crtW* were cut from the plasmids by the *Not*I enzyme. Refer to the system of in vitro recombination, the 50 μL system of *crtZ* and *crtW* inserted randomly in genome contained fragments of *crtZ* and *crtW* from different sources (1000 ng, respectively). Then the fragments pool was transformed into the hosts yQDD001. SC-Leu-Ura + G418 or SC-Leu-His + G418 and SC-Leu-Ura-His + G418 medium are used to select for recombined constructs. To ensure the quality of color screening, all the yeast transformation was diluted fivefold and repeated three times in this study. There are thousands of colonies generated for screening high producing strains.

### Shake flask cultivation for astaxanthin production

For shake flask culture, recombinant yeast colonies were inoculated into 5 mL SC-Leu + G418, SC-Leu-Ura + G418, SC-Leu-His + G418, or SC-Leu-Ura-His + G418 liquid medium respectively at 250 r.p.m., 30 °C for 24 h. Then the preculture was inoculated into the corresponding fresh SC defective medium (50 mL) with an initial OD600 of 0.2 for further 14 h cultivations (OD600 ≈ 5.0). Then seed culture was transferred into 50 mL fresh YPD-40 medium (40 g L^−1^ glucose, 20 g L^−1^ tryptone and 10 g L^−1^ yeast extract) at an initial OD600 of 0.1 grown for 84 h with the condition of 250 r.p.m., 30 °C. Each sample was performed on technical triplicates.

### Growth curve assay

The single colonies were cultured to saturation in 5 mL YPD medium at 30 °C. The cultures were inoculated into a 250 mL shake flask containing 50 mL of YPD medium with initial OD_600_ at 0.1, and cultured at 30 °C, 220 rpm. The OD value was measured at appropriate intervals. Growth curves were plotted using Origin software.

### Analysis of astaxanthin production by HPLC

1 mL of the saturated culture was centrifuged for 2 min at 12,000*g*. Cells were washed with 1 mL ddwater twice and resuspended in 1 mL of 3M HCl. The resuspended cells were heated in the boiling water bath for 2 min and then cooled in ice-bath for 3 min, repeating three times. Then the samples were washed twice with ddwater to wash out HCl and harvested by centrifugation at 12,000*g* for 2 min. After removal of the supernatant, the cells were resuspended in 500 μL acetone and vortexed for 20 min. Acetone extracts were centrifuged (13,000*g*, 15 min) and filtered into a 0.22 μm filter for subsequent. Astaxanthin yield of samples was determined by HPLC (Waters 2695) equipped with HyPURTY C18 column (150 mm × 4.6 mm, Thermo Scientific) and UV detection at 450 nm and 470 nm at 25 °C. The following two buffers were used: A buffer, acetonitrile/water (9:1 vol/vol) and B buffer, methanol/2-propanol (3:2 vol/vol). The flow rate of the mobile phase was 1 mL min^−1^, and the solvent gradient were as follows: from 0 to 15 min for 100% to 10% of A buffer and 0% to 90% of B buffer, and then from 16 to 30 min for 10% of A buffer and 90% of B buffer, then from 31 to 35 min for 10% to 100% of A buffer and 90% to 0% of B buffer, at last from 35 to 55 min for 100% of A and 0% of B buffer. Each sample was performed on technical triplicates.

### PCRtag analysis

15 μL PCR reaction systems contained 7.5 μL 2× rapid Taq master mix (Vazyme), 0.3 μL forward primer (10 μM), 0.3 μL reverse primer (10 μM), 1 μL genome DNA, and 4.9 μL ddH2O. The procedure: 95 °C/3 min, 30 cycles of (95 °C/15 s, 53 °C/30 s, 72 °C/15 s), and 72 °C/5 min. Agarose gel electrophoresis was used for PCR analysis. All primers used in this study were listed in Additional file 1: Table S2.

### Screening and verification of selected strains

For preliminary screening, darker red and big colonies were selected on selective media. Candidate strains were verified on SD media (synthetic complete medium with 20 g L^−1^ glucose) using a tenfold serial dilution assay.

### Extraction of the yeast genomic DNA

Strains were cultured overnight to saturation. Centrifuged at 12,000 rpm to harvest cells. 200 μL breaking buffer (500 mM L^−1^ NaCl, 200 mM L^−1^ Tris–HCl, 100 mM L^−1^ EDTA, 1% SDS), 200 μL silica sand and 200 μL phenol/chloroform/isoamyl alcohol (25:24:1) were added to cells tube. Disrupted cells by the vortex mixer for 20 min. Then added 1 mL cold ethanol to the supernatant, mixed and centrifuged at 4 °C for 10 min. The precipitate was washed with 75% cold ethanol and dried at 37 °C. 200 μL ddH_2_O was added to dissolve the yeast genome DNA. Stored the genome DNA at − 20 °C.

### Quantitative real-time PCR (qPCR) analysis

qPCR was applied to quantify copy numbers of the gene in engineered strains. The template for the qPCR analysis was yeast genomic DNA. Reference primers were selected from gene ALG9 and the target primers were selected from the cassettes of *crtZ* and *crtW* respectively. Strain with a single copy of *crtZ* and *crtW* was used as the reference strain. The copy numbers were determined by comparing the Ct values of *crtZ* or *crtW* and the reference gene ALG9 using the 2^−ΔΔCt^ method. The relative ratio of *crtZ* or *crtW* was calculated as 2^−Δt(crtZ)^ or 2^−ΔCt(crtW)^. Unique Aptamer TM qPCR SYBR Green Master Mix (Beijing Novogene Bioinformatics Technology Co., Ltd) was used for the qPCR reaction, and the equipment was Quantagene q225 (Novogene). The reaction procedure was performed as follows: precycling, 95 °C/300 s, 40 cycles of (95 °C/10 s, 57 °C/20 s, 72 °C/20 s), melt curve, which started from 60 to 95 °C.

## Supplementary information

**Additional file 1: Figure S1.** HPLC analysis of the yQDD000 and yQDD001. a Beta-carotene producing strain yQDD000 showed an onefold β-carotene peak at 20.2 min. b Strain yQDD001 showed astaxanthin peak at 6.4 min along with other peaks for the identified intermediates, such as zeaxanthin (IV) at 7.5 min, canthaxanthin (III) at 10.5 min and lycopene (II) at 18.3 min. **Figure S2.** Principle of the in vitro recombination. Marker for Hygromycin, *Ura3* and *His3* were used to screen the acceptor vector, *crtZ* and *crtW* donor vector respectively. Then acceptor fragments and donor fragments with LoxPSym sites were exposed by restriction enzyme digestion, producing the in vitro recombination reaction pool. Next the pool of diverse plasmids was produced by the action of Cre recombinase. Reaction pool was transformed to target yeast and screening for the yeast library with different astaxanthin yield. The darker red colonies were selected for genotype and phenotype assay. **Figure S3.** Copy number analysis of *crtZ* and *crtW* in strains that selected from in vitro recombination. a The *crtZ* copies number analysis of yQDD002, yQDD003, yQDD004, yQDD005, yQDD009, yQDD010. b The *crtW* copies number analysis of yQDD006, yQDD007, yQDD008, yQDD009, yQDD010. The results indicated that there have only one copy of *crtZ* or *crtW* in the yQDD002-yQDD010. **Figure S4.** Homologous arm design of *in vivo* recombination. Ty1 retrotransposon site is consisted of TyA and TyB. And there are two δ sites on the each side of Ty1 site. Two homologous arms of Ty1 (Ty-1 and Ty-2) were integrated into the flank of the *crtZ*/*crtW* fragment. **Figure S5.** Principle of the *in vivo* recombination. The DNA fragments of *in vivo* recombination were linearized by the digestion of *Not*I. Then the pool of all the DNA fragments was transformed into yQDD001, generating the yeast library with different color. The darker red colonies were selected for genotype and phenotype assay. **Figure S6.** Copy number analysis of *crtZ* and *crtW* in strains that selected from *in vivo* recombination. a The copies number analysis of *crtZ* in yQDD011, yQDD012, yQDD013, yQDD017, yQDD018, yQDD019, yQDD020, yQDD021 and yQDD022. b The copy number analysis of *crtW* in yQDD014, yQDD015, yQDD016, yQDD017, yQDD018, yQDD019, yQDD020, yQDD021, yQDD022. **Figure S7.** Transformation efficiency comparison between the in vitro and the *in vivo* recombination. The colony’s number after yeast transformation with three biological repeats was used to assess the efficiency of the in vitro and the *in vivo* recombination. All the experimental conditions remained consistent, including the concentration of DNA fragment of *crtZ* and *crtW*, the biomass of host strain yQDD001 and other operating environments. Photograph of the in vitro and the in *vivo* screening was attached. The colony’s number was listed in Fig. S6. Compared with in vitro recombination, the much more colonies of *in vivo* screening indicating the high transformation efficiency of the *in vivo* recombination. **Figure S8.** Color stability assay of yQDD008 and yQDD022. The yQDD008 and yQDD022 were serially subcultured in YPD for 6 days and screened on SD agar each day. **Figure S9.** Profile of glucose titer during fermentation in 250-mL flasks with strain yQDD008 and yQDD022. The profile of glucose titer during fermentation was similar between yQDD008 and yQDD022. **Table S1.** PCRTag used in this study. **Table S2.** Primers used in this study.

## Data Availability

All data generated or analyzed during this study are included in this article.

## Abbreviations

*crtW*: β-carotene ketolase
*crtZ*: β-carotene hydroxylation
HPLC: High-pressure liquid chromatography
