# Supplementary material for: In vitro and in vivo recombination of heterologous modules for improving biosynthesis of astaxanthin in yeast
Source: Microb Cell Fact. 2020 May 12;19:103. doi: 10.1186/s12934-020-01356-7 (PMC7216642; doi:10.1186/s12934-020-01356-7)
Supplement: Supplementary file 1 — Additional file 1: Figure S1. HPLC analysis of the yQDD000 and yQDD001. a Beta-carotene producing strain yQDD000 showed an onefold β-carotene peak at 20.2 min. b Strain yQDD001 showed astaxanthin peak at 6.4 min along with other peaks for the identified intermediates, such as zeaxanthin (IV) at 7.5 min, canthaxanthin (III) at 10.5 min and lycopene (II) at 18.3 min. Figure S2. Principle of the in vitro recombination. Marker for Hygromycin, Ura3 and His3 were used to screen the acceptor vector, crtZ and crtW donor vector respectively. Then acceptor fragments and donor fragments with LoxPSym sites were exposed by restriction enzyme digestion, producing the in vitro recombination reaction pool. Next the pool of diverse plasmids was produced by the action of Cre recombinase. Reaction pool was transformed to target yeast and screening for the yeast library with different astaxanthin yield. The darker red colonies were selected for genotype and phenotype assay. Figure S3. Copy number analysis of crtZ and crtW in strains that selected from in vitro recombination. a The crtZ copies number analysis of yQDD002, yQDD003, yQDD004, yQDD005, yQDD009, yQDD010. b The crtW copies number analysis of yQDD006, yQDD007, yQDD008, yQDD009, yQDD010. The results indicated that there have only one copy of crtZ or crtW in the yQDD002-yQDD010. Figure S4. Homologous arm design of in vivo recombination. Ty1 retrotransposon site is consisted of TyA and TyB. And there are two δ sites on the each side of Ty1 site. Two homologous arms of Ty1 (Ty-1 and Ty-2) were integrated into the flank of the crtZ/crtW fragment. Figure S5. Principle of the in vivo recombination. The DNA fragments of in vivo recombination were linearized by the digestion of NotI. Then the pool of all the DNA fragments was transformed into yQDD001, generating the yeast library with different color. The darker red colonies were selected for genotype and phenotype assay. Figure S6. Copy number analysis of crtZ and crtW in strains that [file 12934_2020_1356_MOESM1_ESM.docx]

**Additional file**

*In vitro* and *In vivo* recombination of heterologous modules for improving biosynthesis of astaxanthin in yeast

Dan-Dan Qi^1, 2^, Jin Jin^1, 2^, Duo Liu^1, 2^, Bin Jia ^1, 2*^ and Ying-Jin Yuan ^1, 2^

^1^Frontier Science Center for Synthetic Biology and Key Laboratory of Systems Bioengineering (Ministry of Education), Tianjin University, Tianjin 300072, China

^2^Collaborative Innovation Center of Chemical Science and Engineering (Tianjin), School of Chemical Engineering and Technology, Tianjin University, Tianjin 300072, China

* To whom all correspondence should be addressed:

Dr. Bin Jia

Email: bin.jia@tju.edu.cn

Email addresses for all authors: [2017207246@tju.edu.cn](mailto:2017207246@tju.edu.cn);[2015207264@tju.edu.cn](mailto:2015207264@tju.edu.cn); [liuduo19870401@126.com；bin.jia@tju.edu.cn](mailto:liuduo19870401@126.com；bin.jia@tju.edu.cn); [yjyuan@tju.edu.cn](mailto:yjyuan@tju.edu.cn).

**Figures**


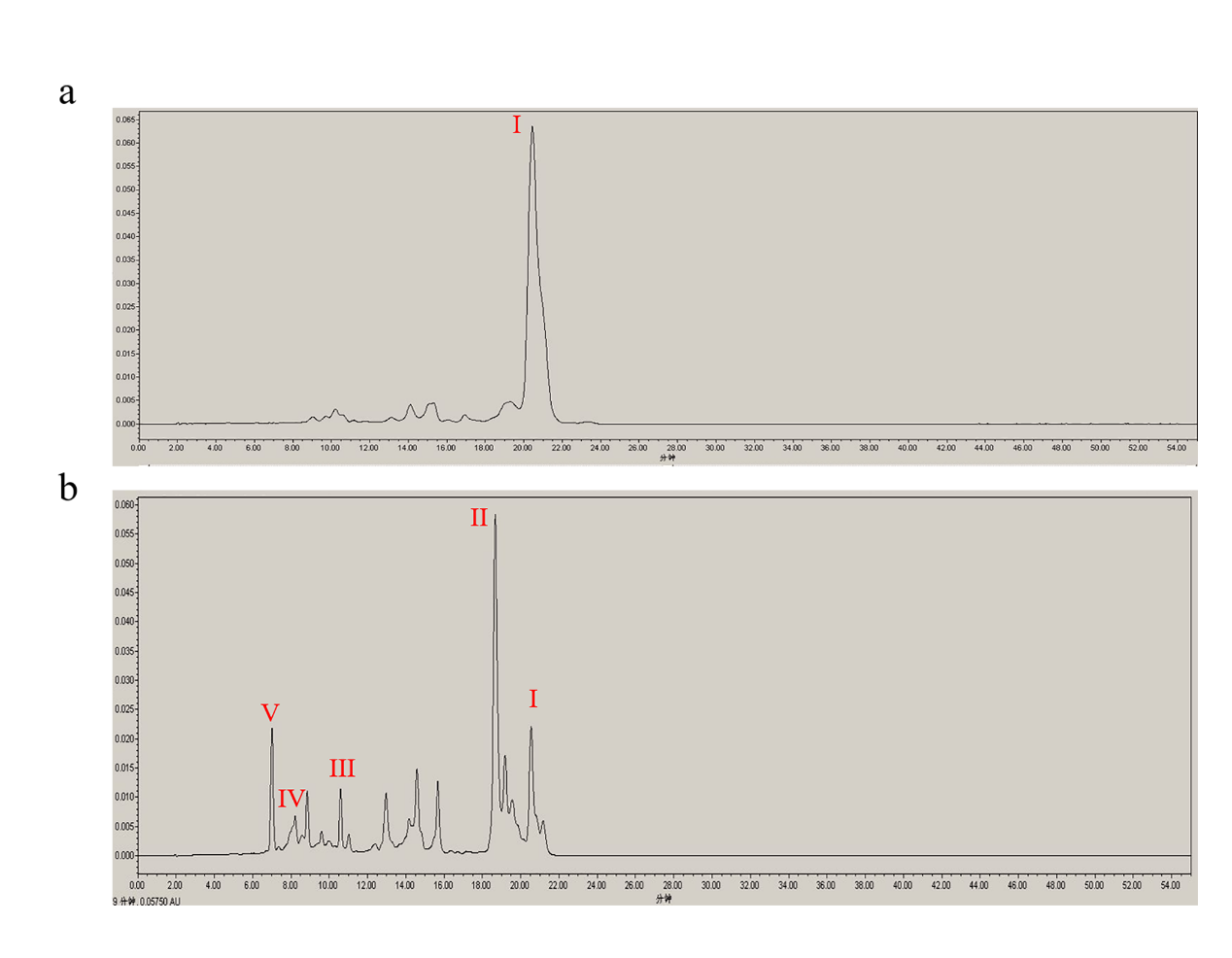


**Fig. S1 HPLC analysis of the yQDD000 and yQDD001. a** Beta-carotene producing strain yQDD000 showed an onefold β-carotene peak at 20.2 min. **b** strain yQDD001 showed astaxanthin peak at 6.4 min along with other peaks for the identified intermediates, such as zeaxanthin (IV) at 7.5 min, canthaxanthin (III) at 10.5 min and lycopene (II) at 18.3 min.

**
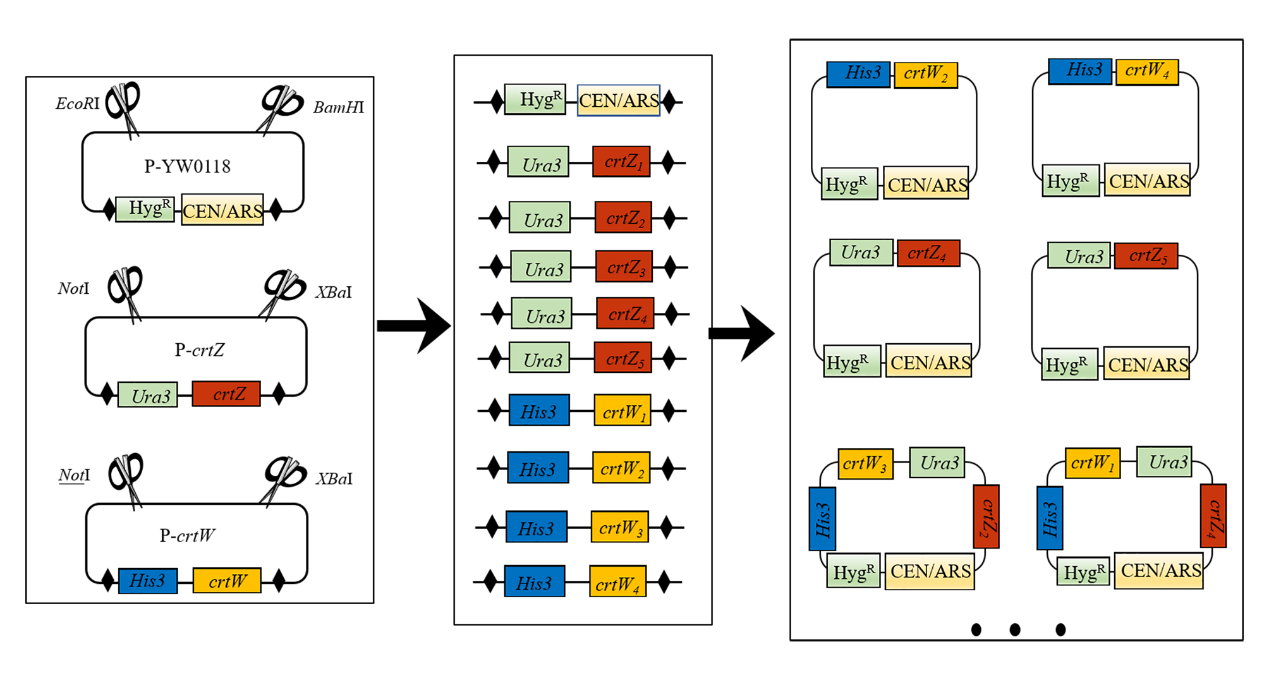
 Fig. S2 Principle of the *in vitro* recombination.** Marker for Hygromycin, *Ura3* and *His3* were used to screen the acceptor vector, *crtZ* and *crtW* donor vector respectively. Then acceptor fragments and donor fragments with loxpsym sites were exposed by restriction enzyme digestion, producing the *in vitro* recombination reaction pool. Next the pool of diverse plasmids was produced by the action of Cre recombinase. Reaction pool was transformed to target yeast and screening for the yeast library with different astaxanthin yield. The darker red colonies were selected for genotype and phenotype assay.

**
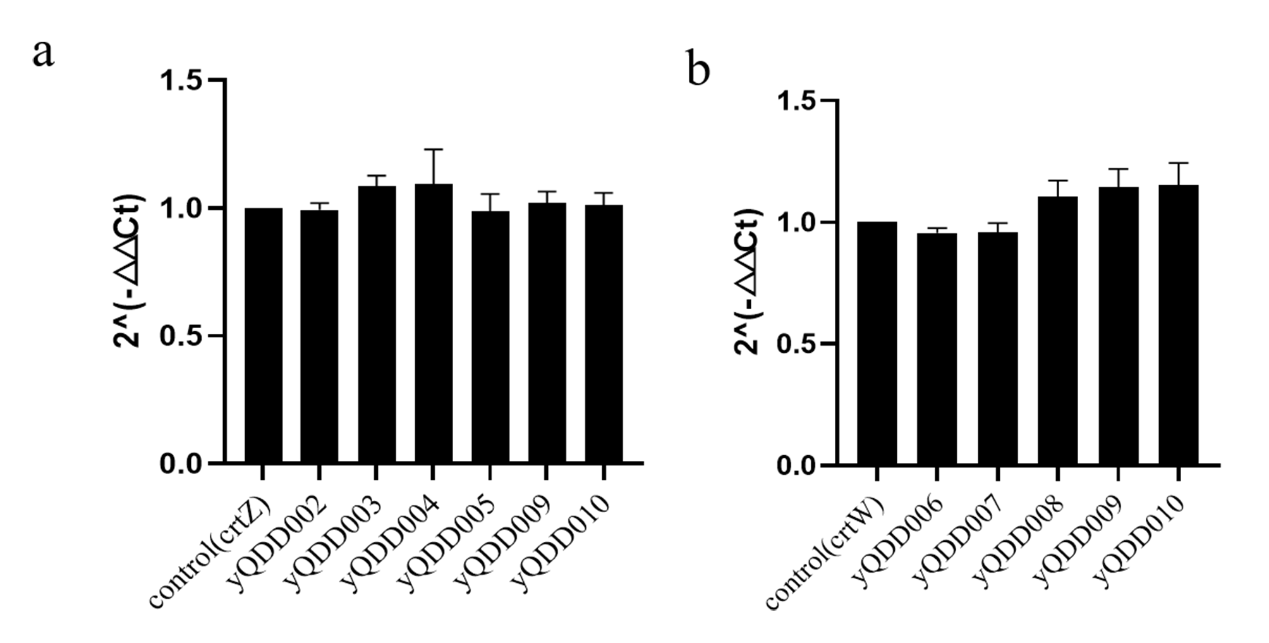
Fig. S3 Copy number analysis of *crtZ* and *crtW* in strains that selected from *in vitro* recombination. a** The *crtZ* copy number analysis of yQDD002, yQDD003, yQDD004, yQDD005, yQDD009, yQDD010. **b** The *crtW* copy number analysis of yQDD006, yQDD007, yQDD008, yQDD009, yQDD010.The results indicated that there have only one copy of *crtZ* or *crtW* in the yQDD002-yQDD010.


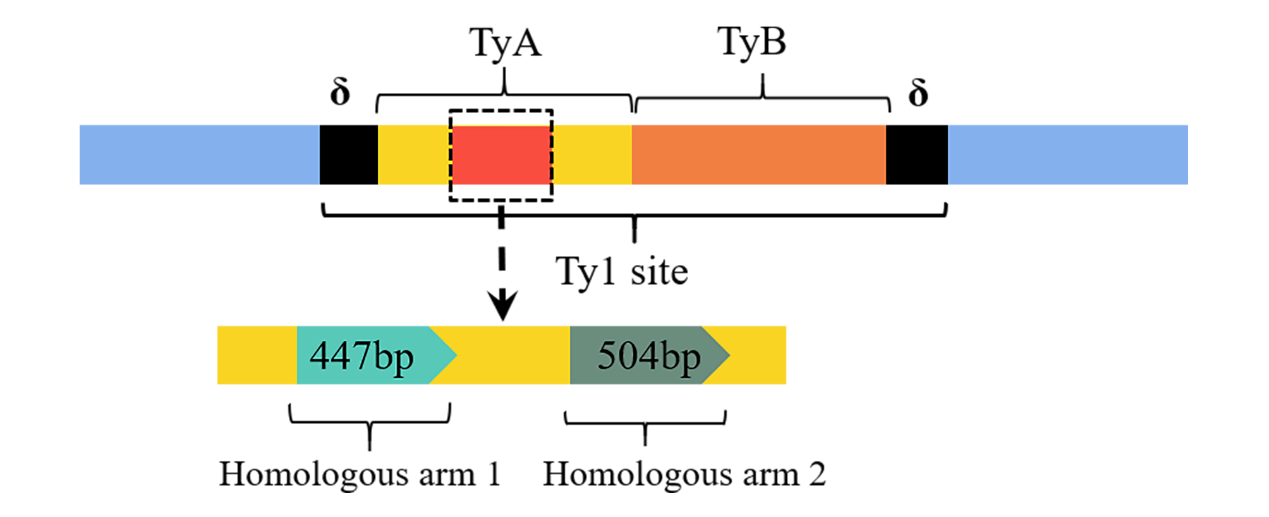


**Fig. S4 Homologous arm design of *in vivo* recombination.** Ty1 retrotransposon site is consisted of TyA and TyB. And there are two δ sites on the each side of Ty1 site. Two homologous arms of Ty1 (Ty-1 and Ty-2) were integrated into the flank of the *crtZ*/*crtW* fragment.


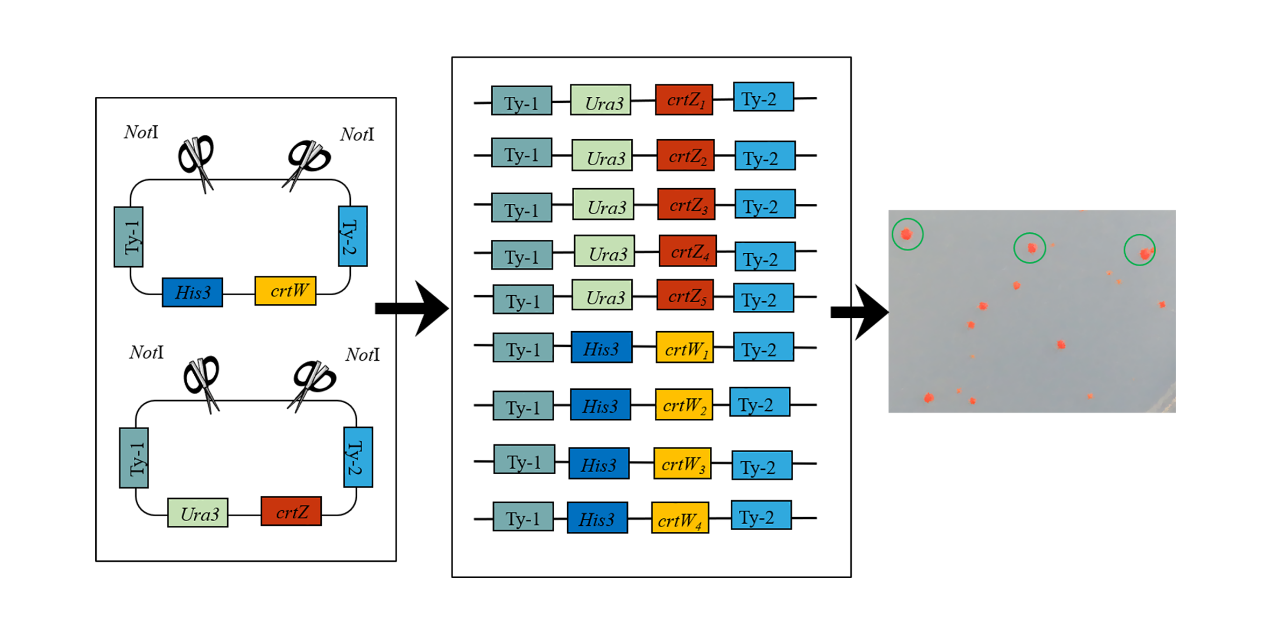


**Fig. S5 Principle of the *in vivo* recombination.** The DNA fragments of *in vivo* recombination were linearized by the digestion of *Not*I. Then the pool of all the DNA fragments was transformed into yQDD001, generating the yeast library with different color. The darker red colonies were selected for genotype and phenotype assay.

**
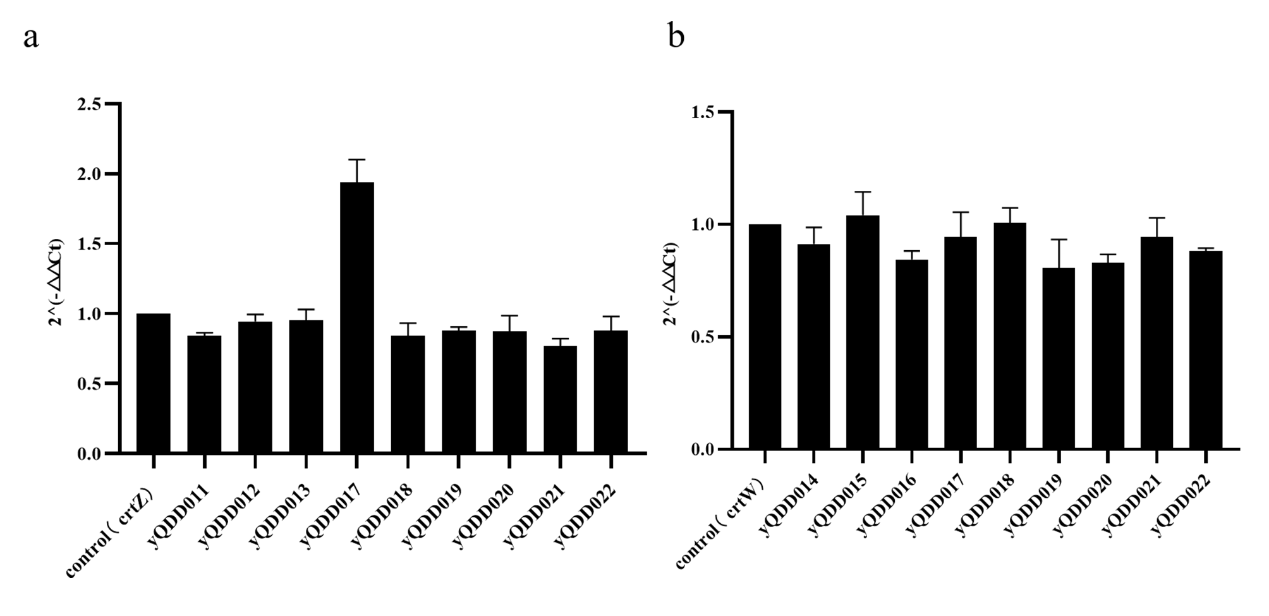
**

**Fig. S6 Copy number analysis of *crtZ* and *crtW* in strains that selected from *in vivo* recombination. a** The *crtZ* copy number analysis of yQDD011, yQDD012, yQDD013, yQDD017, yQDD018, yQDD019, yQDD020, yQDD021 and yQDD022. **b** The *crtW* copy number analysis of yQDD014, yQDD015, yQDD016, yQDD017, yQDD018, yQDD019, yQDD020, yQDD021, yQDD022.


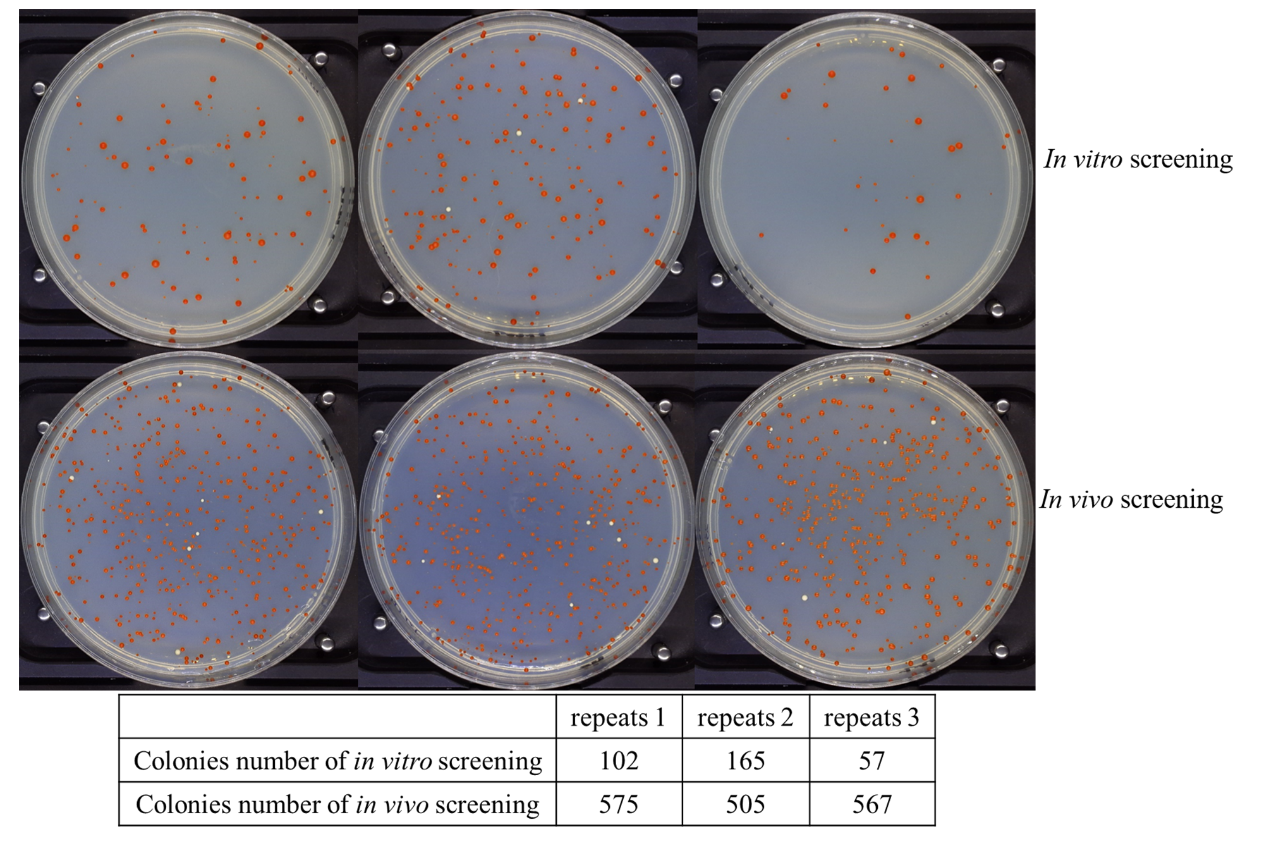


**Fig. S7 Transformation efficiency comparison between the *in vitro* and the *in vivo* recombination.** The colony's number after yeast transformation with three biological repeats was used to assess the efficiency of the *in vitro* and the *in vivo* recombination. All the experimental conditions remained consistent, including the concentration of DNA fragment of *crtZ* and *crtW*, the biomass of host strain yQDD001 and other operating environments. Photograph of the *in vitro* and the in *vivo* screening was attached. The colony's number was listed in Fig. S7. Compared with the *in vitro* recombination, the much more colonies of *in vivo* screening indicating the high transformation efficiency of the *in vivo* recombination.


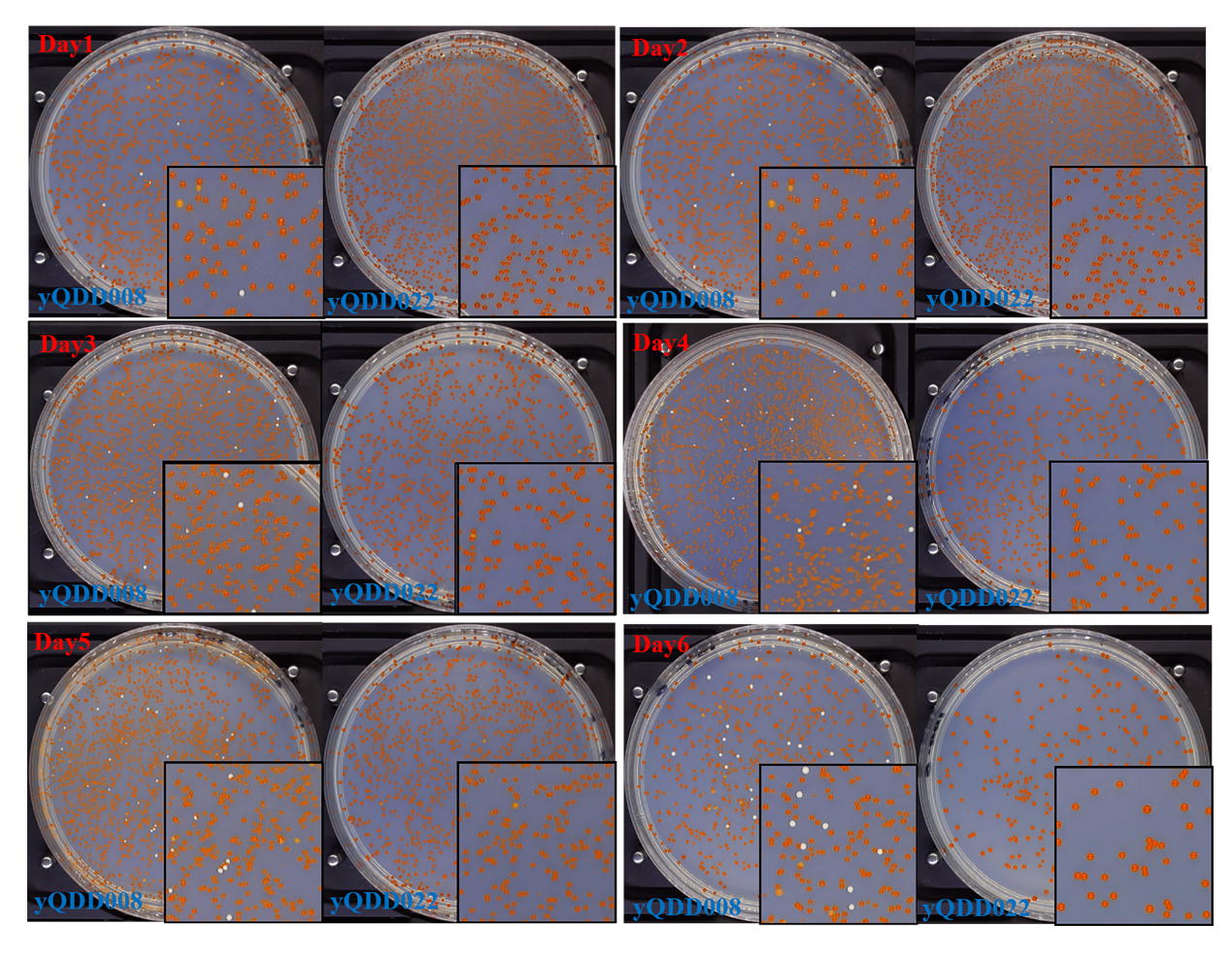


**Fig. S8 Color stability assay of yQDD008 and yQDD022.** The yQDD008 and yQDD022 were serially subcultured in YPD for 6 days and screened on SD agar each day.

**
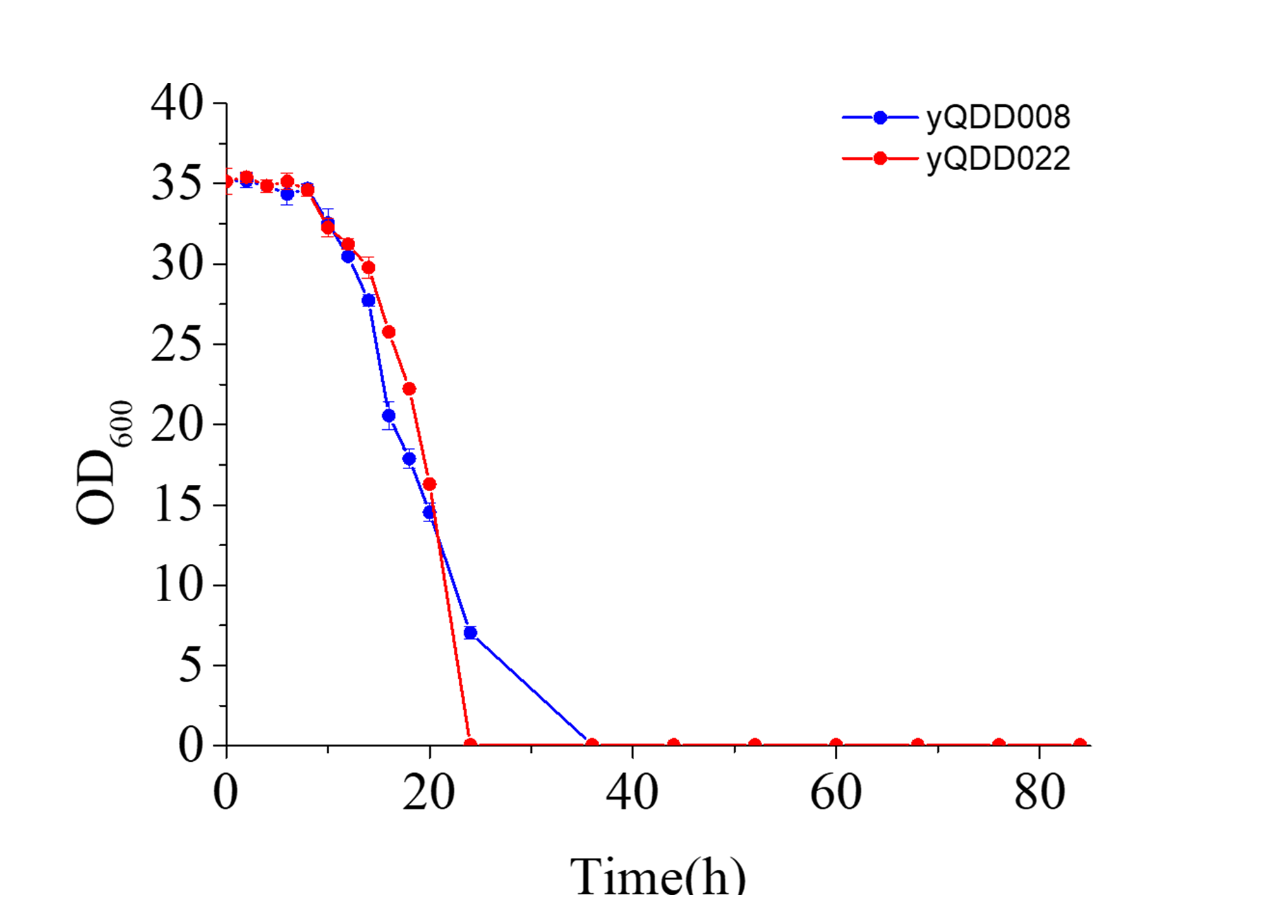
Fig. S9** **Profile of glucose titer during fermentation in 250-mL flask with strain yQDD008 and yQDD022.** The profile of glucose titer during fermentation was similar between yQDD008 and yQDD022

**Tables**

**Table S1** PCRTag used in this study

| Name | Sequences |
| --- | --- |
| tag1 | ACAAGGATTTTTGACGGTGC |
| tag2 | AAAGCATCGTAGCACTGCTC |
| tag3 | GAGCCTTTGATCGCAATCTT |
| tag4 | CGAGCGACAGACTGGTATTC |
| tag5 | GTCGTTGAGTACCGGCATCA |
| tag6 | GGCTGAGATCAAAGGCGGTT |
| tag7 | CGGCAAAGCTGGTTTGAATT |
| tag8 | GATATCGTTGAGCAGCTTGG |
| tag9 | CATTTGGTACTGGCCAGACC |

**Table S2** Primers used in this study

| Name | Sequences | Description |
| --- | --- | --- |
| Z_1_+1pcrtag-R | CGCTCTTATTGACCACACCTCGCACCGTCAAAAATCCTTGT | Amplification of *crtZ_1_* with 1PCRTag |
| Z_1_+1pcrtag-F | GCACCGTCAAAAATCCTTGTTACCGGCATGTCAATTCATCAT | Amplification of *crtZ_1_* with 1PCRTag |
| Z_2_+2pcrtag-R | CGCTCTTATTGACCACACCTCGAGCAGTGCTACGATGCTTT | Amplification of *crtZ_2_* with 2PCRTag |
| Z_2_+2pcrtag-F | GAGCAGTGCTACGATGCTTTTACCGGCATGTCAATTCATCAT | Amplification of *crtZ_2_* with 2PCRTag |
| Z_3_+3pcrtag-R | CGCTCTTATTGACCACACCTCAAGATTGCGATCAAAGGCTC | Amplification of *crtZ_3_* with 3PCRTag |
| Z_3_+3pcrtag-F | AAGATTGCGATCAAAGGCTCTACCGGCATGTCAATTCATCAT | Amplification of *crtZ_3_* with 3PCRTag |
| Z_4_+4pcrtag-R | CGCTCTTATTGACCACACCTCGAATACCAGTCTGTCGCTCG | Amplification of *crtZ_4_* with 4PCRTag |
| Z_4_+4pcrtag-F | GAATACCAGTCTGTCGCTCGTACCGGCATGTCAATTCATCAT | Amplification of *crtZ_4_* with 4PCRTag |
| Z_5_ +5pcrtag-R | CGCTCTTATTGACCACACCTCTGATGCCGGTACTCAACGAC | Amplification of *crtZ_5_* with 5PCRTag |
| Z_5_ +5pcrtag-F | TGATGCCGGTACTCAACGACTACCGGCATGTCAATTCATCAT | Amplification of *crtZ_5_* with 5PCRTag |
| W_1_+6pcrtag-R | CACACTAATTGGCTTTTCGCAACCGCCTTTGATCTCAGCC | Amplification of *crtW_1_* with 6PCRTag |
| W_1_+6pcrtag-F | AACCGCCTTTGATCTCAGCCATTGGTGAGCGCTAGGAG | Amplification of *crtW_1_* with 6PCRTag |
| W_2_ +7pcrtag-R | CACACTAATTGGCTTTTCGCAATTCAAACCAGCTTTGCCG | Amplification of *crtW_2_* with 7PCRTag |
| W_2_ +7pcrtag-F | AATTCAAACCAGCTTTGCCGATTGGTGAGCGCTAGGAG | Amplification of *crtW_2_* with 7PCRTag |
| W_3_+8pcrtag-R | CACACTAATTGGCTTTTCGCCCAAGCTGCTCAACGATATC | Amplification of *crtW_3_* with 8PCRTag |
| W_3_+8pcrtag-F | CCAAGCTGCTCAACGATATCATTGGTGAGCGCTAGGAG | Amplification of *crtW_3_* with 8PCRTag |
| W_4_+9pcrtag-R | CACACTAATTGGCTTTTCGCGGTCTGGCCAGTACCAAATG | Amplification of *crtW_4_* with 9PCRTag |
| W_4_+9pcrtag-F | GGTCTGGCCAGTACCAAATGATTGGTGAGCGCTAGGAG | Amplification of *crtW_4_* with 9PCRTag |
| Z-F | TGGTAGATACGTTGTTGACACTTC | PCRTag analysis of *crtZ* |
| w-F | ACCTGAAGCATAACTGACACTAC | PCRTag analysis of *crtW* |
| tag1-R | ACAAGGATTTTTGACGGTGC | PCRTag analysis |
| tag2-R | AAAGCATCGTAGCACTGCTC | PCRTag analysis |

**Table S2** Primers used in this study (continued)

| Name | Sequences | Description |
| --- | --- | --- |
| tag3-R | GAGCCTTTGATCGCAATCTT | PCRTag analysis |
| tag4-R | CGAGCGACAGACTGGTATTC | PCRTag analysis |
| tag5-R | GTCGTTGAGTACCGGCATCA | PCRTag analysis |
| tag6-R | GGCTGAGATCAAAGGCGGTT | PCRTag analysis |
| tag7-R | CGGCAAAGCTGGTTTGAATT | PCRTag analysis |
| tag8-R | GATATCGTTGAGCAGCTTGG | PCRTag analysis |
| tag9-R | CATTTGGTACTGGCCAGACC | PCRTag analysis |
| Hph-F | CGAAGTTATCCCAGGTCGCTCGTCCCAAAACCTTCTCAAGC | Amplification of Hyg |
| Hph-R | CCACCGCGGTGGCGGCCGCTGCATCAGAGCAGATTGTACTGAGAG | Amplification of Hyg |
| Hph-test-1-F | ACTCTTTGATAACGTCTTCGGAGG | Verification for Hyg |
| Hph-test-1-R | TGTAGAAGTACTCGCCGATAGTGG | Verification for Hyg |
| Hph-test-2-F | TGTTCGGATGTGATGTGAGAACTG | Verification for Hyg |
| Hph-test-2-R | CGAACTGAGATACCTACAGCGT | Verification for Hyg |
| *crtW*-F | TGTGACCGTAAAACGACGGCCAGTCAGTTCGAGTTTATCATTATCAATAC | Amplification of *crtW* |
| *crtW*-R | AATGCATCAGGAAACAGCTATGACCTACATAAGAACACCTTTGGTGGAGG | Amplification of *crtW* |
| *crtZ*-F | TTGTGACCGTAAAACGACGGCCAGTTCCAACTGGCACCGCTGGCTTGAAC | Amplification of *crtZ* |
| *crtZ*-R | AAATGCATCAGGAAACAGCTATGACTTAGTTTTGCTGGCCGCATCTTCT | Amplification of *crtZ* |
| Ty-F | GTCATAGCTGTTTCCTGATGCATTTGAAACAAAAGTCACAAAC | Amplification of Ty homologous arm |
| Ty-R | ACTGGCCGTCGTTTTACGGTCACAATGTCGTTTGCCTCT | Amplification of Ty homologous arm |
